# A brain-inspired memory transformation based differentiable neural computer for reasoning-based question answering

**DOI:** 10.3389/frai.2025.1635932

**Published:** 2025-08-14

**Authors:** Yao Liang, Yuwei Wang, Hongjian Fang, Feifei Zhao, Yi Zeng

**Affiliations:** ^1^Brain-inspired Cognitive Intelligence Lab, Institute of Automation, Chinese Academy of Sciences, Beijing, China; ^2^School of Artificial Intelligence, University of Chinese Academy of Sciences, Beijing, China; ^3^Center for Long-term Artificial Intelligence, Beijing, China; ^4^School of Future Technology, University of Chinese Academy of Sciences, Beijing, China; ^5^Key Laboratory of Brain Cognition and Brain-inspired Intelligence Technology, Chinese Academy of Sciences, Shanghai, China

**Keywords:** neural turing machine, memory-augmented networks, reasoning and question answering, working/long-term memory, differentiable neural computer

## Abstract

Reasoning and question answering, as fundamental cognitive functions in humans, remain significant hurdles for artificial intelligence. While large language models (LLMs) have achieved notable success, integrating explicit memory with structured reasoning capabilities remains a persistent difficulty. The Differentiable Neural Computer (DNC) model, despite addressing these issues to some extent, still faces challenges such as algorithmic complexity, slow convergence, and limited robustness. Inspired by the brain's learning and memory mechanisms, this paper proposes a Memory Transformation based Differentiable Neural Computer (MT-DNC) model. The MT-DNC integrates two brain-inspired memory modules—a working memory module inspired by the cognitive system that temporarily holds and processes task-relevant information, and a long-term memory module that stores frequently accessed and enduring information—within the DNC framework, enabling the autonomous transformation of acquired experiences between these memory systems. This facilitates efficient knowledge extraction and enhances reasoning capabilities. Experimental results on the bAbI question answering task demonstrate that the proposed method outperforms existing Deep Neural Network (DNN) and DNC models, achieving faster convergence and superior performance. Ablation studies further confirm that the transformation of memory from working memory to long-term memory is critical for improving the robustness and stability of reasoning. This work offers new insights into incorporating brain-inspired memory mechanisms into dialogue and reasoning systems.

## 1 Introduction

Reasoning and Question Answering (QA) are fundamental cognitive functions that are central to evaluating artificial intelligence systems. Despite the remarkable success of large language models (LLMs) (Touvron et al., 2023; Dubey et al., 2024; Achiam et al., 2023), challenges remain in developing methods that integrate explicit memory and structured reasoning capabilities. The Differentiable Neural Computer (DNC) model, proposed by (Graves et al. 2016), provides a feasible solution for studying reasoning and QA. DNC consists of a DNN-based computational controller and an external memory module, with which the neural network can interact (read and write). The memory module is responsible for representing and storing learned structures.

The DNC model has demonstrated good performance on various image reasoning and QA tasks (Graves et al., 2016; Rasekh and Safi-Esfahani, 2020). However, it faces several key challenges, including high algorithmic complexity, slow convergence speed, and a high average test error rate, all of which limit its further development and broader application. The BrsDNC model (Franke et al., 2018) improves the DNC model by introducing normalization and dropout, which have been shown to enhance robustness and scalability. The primary issues with current DNC models stem from restricted memory, which may lead to the loss of critical knowledge. As training time increases, the pressure on the memory module for reading and writing grows rapidly, thus limiting the model's training speed and performance. Besides, existing methods lack references from brain learning and memory mechanisms. Thus, there is still much room for improvement.

Memory in the brain encompasses both short-term and long-term memory, among others (Baddeley, 2007; Lee and Wilson, 2002; Winocur et al., 2010; Marshall and Born, 2007; Ji and Wilson, 2007). These types of memory play crucial roles in various cognitive functions, including learning, decision-making, and reasoning. Short-term memory has limited storage capacity and, therefore, cannot retain information indefinitely (Diamond, 2013). As a result, some memories are forgotten, while others that are repeatedly accessed are retained and transferred to long-term memory. Information can be stored in long-term memory for extended periods, continuously aiding learning and reasoning (Atkinson and Shiffrin, 1968). The collaboration and division of labor between working memory and long-term memory enable the brain to consolidate and apply acquired knowledge more efficiently, thereby enhancing the brain's capacity to perform multiple cognitive tasks (Kitamura et al., 2017). While short-term memory refers primarily to the brief retention of information, working memory further includes active manipulation and processing of information required for cognitive tasks, thus making it distinct and crucial for reasoning.

Inspired by the brain's learning and memory mechanisms, we propose a brain-inspired Memory Transformation based Differentiable Neural Computer (MT-DNC). Unlike the original DNC model, which has a single memory module, MT-DNC introduces two distinct memory modules: working memory and long-term memory. Working memory stores information directly relevant to the current task, while long-term memory holds more meaningful, enduring knowledge. These two memory modules are interconnected through a memory transformation algorithm. The core principles of the memory transformation algorithm are as follows: knowledge that is repeatedly accessed is transferred to long-term memory, while irrelevant information is discarded from working memory (Zhao et al., 2017; LeCun et al., 2015).

The innovations of our method are primarily reflected in the following aspects:

1. Integration of working and long-term memory: MT-DNC introduces a novel architecture that explicitly combines working memory and long-term memory. This design enhances the model's ability to comprehensively store and utilize acquired knowledge, mimicking the human brain's memory system.
2. Brain-inspired memory transformation algorithm: A key contribution of MT-DNC is the development of a memory transformation algorithm inspired by biological memory mechanisms. This algorithm dynamically identifies and retains useful information by transferring it from working memory to long-term memory, while discarding irrelevant data, thereby optimizing memory efficiency.
3. Improved performance on reasoning tasks: Extensive experiments on the *bAbI* reasoning-based question-answering benchmark demonstrate that MT-DNC achieves superior accuracy and faster convergence compared to existing DNC-based methods. Moreover, the results highlight the crucial role of memory transformation in enhancing the model's stability and robustness during complex reasoning tasks.

## 2 Related work

**Neural Turing Machine (NTM):** The core idea of NTM is to combine neural networks with external memory, thereby expanding the capabilities of neural networks and enabling interaction through an attention mechanism (Graves et al., 2014). To some extent, NTM can be compared to a Turing machine (Xiong et al., 2016; Zaremba and Sutskever, 2015), with experiments verifying its Turing completeness (Tao et al., 2021; Zaremba and Sutskever, 2015). The main advantage of NTM is its ability to handle complex tasks that require memory participation.

**Differentiable Neural Computer (DNC):** DNC, which is considered an improved version of NTM, shares the same core idea of using external memory to enhance the ability of neural networks (Graves et al., 2016; Santoro et al., 2016; Lake et al., 2017). Compared to the original NTM, DNC introduces significant improvements in the addressing mechanism (Hassabis et al., 2017; Chan et al., 2018), removes the index shift operation, and better supports memory allocation and de-allocation functions. Additionally, DNC shows notable performance improvements over NTM.

Recent works have further enhanced the DNC architecture. (Franke et al. 2018) improved the model's performance by optimizing the memory module, increasing the bidirectional connections between memory modules, and introducing the layer normalization training method (Ba J. L. et al., 2016). By refining the addressing and memory allocation processes, (Csordás and Schmidhuber 2019) achieved better accuracy on the bAbI task. (Rasekh and Safi-Esfahani 2020) integrated the NeuroEvolution algorithm into the DNC framework, demonstrating faster encoding speed in various cognitive tasks, leading to improved model performance.

To summarize, none of these approaches fully address the issues of low accuracy and slow convergence associated with DNC's limited external memory. This paper draws inspiration from the brain's learning and memory mechanisms and proposes the MT-DNC model, which integrates two coordinated memory modules: working memory and long-term memory (Seo et al., 2016; Ba J. et al., 2016; Le et al., 2019, 2020). The proposed model improves both accuracy and convergence speed, offering superior performance compared to existing DNC-based models.

## 3 Method

In this section, we provide a comprehensive introduction to the MT-DNC model. MT-DNC extends the memory module of the DNC by incorporating both a working memory module and a long-term memory module. Inspired by the brain's learning and memory mechanisms, MT-DNC introduces a dual-memory architecture that consists of both working memory and long-term memory. This architecture enables the model to manage and store information more effectively, thereby enhancing its reasoning and knowledge retention capabilities. The core innovation lies in a dynamic memory transformation mechanism that selectively transfers frequently accessed or meaningful information from working memory to long-term memory, enabling the model to maintain a compact yet informative working memory.

In the MT-DNC architecture, working memory (or short-term memory) rapidly processes and updates information needed immediately, while the long-term memory persistently retains valuable knowledge, with the memory transformation mechanism dynamically managing information transfer between these memory modules to enhance reasoning efficiency.

**Figure 1:**
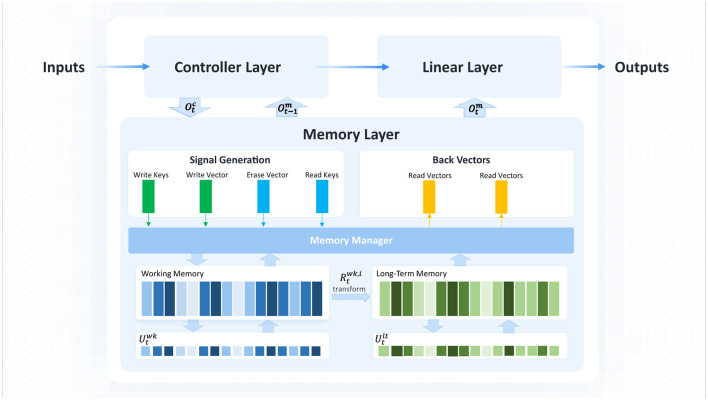
Overall architecture of MT-DNC.

The overall framework of MT-DNC consists of three layers: the controller layer, memory layer, and linear layer, as shown in Figure 1. The controller layer is responsible for encoding and processing both the input data and the output from the previous time step of the controller layer and the memory layer, learning temporal patterns from the training data, and transmitting the results to both the memory and linear layers. The memory layer is responsible for storing the controller's output and extracting useful information through a series of storage and transformation mechanisms. This layer also incorporates memory transformation between the working memory and long-term memory modules, enabling the MT-DNC model to exhibit strong memory and reasoning capabilities. The linear layer combines the outputs from the controller and memory layers, and produces the final prediction result via a linear transformation.

### 3.1 Controller layer

The controller layer combines the original input data $x_{t}\in {\mathbb{R}}^{X}$ with the output of the memory layer from the previous time step, $O_{t-1}^{m}\in {\mathbb{R}}^{2RW}$, as well as the output of the controller layer from the previous time step, after undergoing Dropout processing. After performing a Long Short-Term Memory (LSTM) operation and applying layer normalization (Klambauer et al., 2017; Franke et al., 2018), the resulting output $O_{t}^{c}$ is transmitted to the memory layer. As shown in Equation 1:


(1) $$\begin{array}{l} O_{t}^{c}=\text{LayerNorm}-\text{LSTM}((x_{t}⊕O_{t-1}^{m}⊕\text{Dropout}(O_{t-1}^{c})),c_{t-1};\\ \;W_{t}^{c},b_{t}^{c}), \end{array}$$

where $O_{t}^{c}\in {\mathbb{R}}^{C}$ denotes the output at time step *t*. The term **c**_*t*−1_ represents the cell state from the previous time step, and $W_{t}^{c}\in {\mathbb{R}}^{\left(X+2RW+C\right)\times C}$ is the weight matrix that maps the input to the gates. Additionally, $b_{t}^{c}\in {\mathbb{R}}^{C}$ is the bias vector associated with the input to the gates, and ⊕ denotes the concatenation of vectors.

Here, *X* represents the dimension of the input data, *C* represents the output dimension of the controller layer, and *W* represents the width of the memory region.

### 3.2 Memory layer

The memory layer consists of the working memory module (functionally analogous to working memory in human cognition, temporarily storing and actively processing task-relevant information), the long-term memory module (storing enduring and frequently accessed knowledge), and the memory transformation mechanism. The working memory module stores the most recent interaction data from the controller layer, while the long-term memory holds frequently used information of high importance that may eventually be discarded by the working memory. Both the working memory and long-term memory require dynamic update and extraction rules to continuously replace stored information. The memory transformation mechanism selectively transfers data from working memory to long-term memory for processing. Finally, the memory layer combines the outputs from the controller layer, working memory, and long-term memory to make decisions.

#### 3.2.1 Working memory module

The working memory module is functionally designed to store interactive information from the controller layer's output in real time, updating and extracting relevant information based on the controller layer's output. Due to storage limitations, we draw inspiration from the memory update and decay mechanisms in the human brain, replacing information that is similar to the current interaction data ($O_{t}^{c}$). Additionally, information that has already been extracted or used is more likely to be replaced in order to retain as much novel information as possible.

The read, write, and gating signals within the memory region are generated from $O_{t}^{c}$ through a linear transformation. Let $S_{t}\in {\mathbb{R}}^{\left(2R+6\right)W+6+4R}$ represent the signal vector at time step *t*, derived via layer normalization, as shown in Equation 2:


(2) $$\begin{array}{l} S_{t}=\text{LayerNormalization}\left(O_{t}^{c}\cdot W_{t}^{s}+b_{t}^{s}\right), \end{array}$$

where $W_{t}^{s}\in {\mathbb{R}}^{C\times \left(\left(2R+6\right)W+6+4R\right)}$ is the weight matrix and $b_{t}^{s}\in {\mathbb{R}}^{\left(\left(2R+6\right)W+6+4R\right)}$ is the bias vector. The dimension of *S*_*t*_ is carefully designed based on the operational needs of both working and long-term memory modules, involving signals for writing, reading, erasing, and gating controls. Specifically, (2*R*+6)*W* represents memory signals corresponding to multiple read/write operations across working and long-term memories, while the additional terms 6 and 4*R* account for scalar gates and strengths. A comprehensive step-by-step derivation is provided in Appendix A.

This normalized signal vector is systematically partitioned into several distinct components, each corresponding to specific memory regions and operational functionalities, ensuring that the total length of all variables matches the dimension of *S*_*t*_.

Initially, the first *W* elements of *S*_*t*_ are designated as the write query signal for the working memory region, denoted by $K_{t}^{wk}\in {\mathbb{R}}^{W}$, while the subsequent *W* elements serve as the write query signal for the long-term memory region, denoted by $K_{t}^{lt}\in {\mathbb{R}}^{W}$. Following these, the next two elements are processed through the oneplus activation function to yield the write scaling factors $\beta_{t}^{wk}\in \mathbb{R}$ and $\beta_{t}^{lt}\in \mathbb{R}$ for the working and long-term memory regions, respectively. The oneplus function is defined as:


$$\text{oneplus}\left(x\right)=1+\text{softplus}\left(x\right)=1+\ln\left(1+e^{x}\right)$$

This function ensures that the scaling factors are strictly positive, facilitating stable and controlled scaling during the write operations.

Subsequently, the next 2*W* elements of *S*_*t*_ are passed through the sigmoid activation function to generate the erase signals $E_{t}^{wk}\in {\mathbb{R}}^{W}$ and $E_{t}^{lt}\in {\mathbb{R}}^{W}$, which facilitate the controlled removal of information within the working and long-term memory regions, respectively. The following 2*W* elements are directly extracted to form the write signals $V_{t}^{wk}\in {\mathbb{R}}^{W}$ and $V_{t}^{lt}\in {\mathbb{R}}^{W}$, enabling the storage of new information.

To regulate weight allocation and the strength of write operations, the subsequent four elements are processed through the sigmoid function to derive the gating scalars $g_{t}^{wk}\in \mathbb{R}$, $g_{t}^{lt}\in \mathbb{R}$, $\gamma_{t}^{wk}\in \mathbb{R}$, and $\gamma_{t}^{lt}\in \mathbb{R}$. These gating scalars modulate the write operations within both the working and long-term memory regions effectively.

For multi-head read operations, the signal vector is further partitioned into components corresponding to each of the *R* read heads. Specifically, the read query signals $K_{t}^{wk,i}\in {\mathbb{R}}^{R\times W}$ and $K_{t}^{lt,i}\in {\mathbb{R}}^{R\times W}$ are extracted for the working and long-term memory regions, respectively. The corresponding read scaling factors $\beta_{t}^{wk,i}\in {\mathbb{R}}^{R}$ and $\beta_{t}^{lt,i}\in {\mathbb{R}}^{R}$ are obtained by applying the oneplus function to the relevant segments of *S*_*t*_. Additionally, the free gating vectors $f_{t}^{wk,i}\in {\mathbb{R}}^{R}$ and $f_{t}^{lt,i}\in {\mathbb{R}}^{R}$ are computed using the sigmoid function, providing flexible control over information retrieval across all read heads.

Here, *W* represents the width of the memory region, and *R* specifies the number of read heads. The regions *wk* and *lt* refer to the working and long-term memory, respectively, while *t* denotes the current time step in the signal processing sequence. The total length of all these variables collectively equals the dimension of *S*_*t*_, which is (2*R*+6)*W*+6+4*R*. This meticulous segmentation of *S*_*t*_ into dedicated variables, each with explicitly defined dimensionalities, ensures efficient and optimized storage and retrieval processes across both memory regions. Consequently, this enhances the overall functionality and performance of the working memory module by enabling precise control and manipulation of information within the system.

**Working Memory Updating Algorithm**. The updating of the working memory is based on the following principles:

1. Delete memory slots with lower usage frequency or longer recency intervals. Specifically, items with the lowest usage value, tracked by the usage vector $U_{t}^{wk}$, are prioritized for deletion. The usage vector is updated at each time step based on previous read and write weights, which progressively reduces the usage value of slots that have not been accessed or updated recently.
2. Delete items after extraction, which corresponds to actively setting low retention values using the free gates ($f_{t}^{wk,i}$), effectively marking them for replacement.
3. Delete memory items whose content is highly similar to newly stored information. The similarity is measured by cosine similarity in content-based addressing.
4. Retain recently updated novel items, identified as slots with recent write operations and relatively higher usage values in the usage vector.

Based on these principles, we update the working memory in real-time according to the dynamic addressing algorithm in Equation 3 (Graves et al., 2016; Hsin, 2016).


(3) $$\begin{array}{l} \;\psi_{t}^{wk}=\prod_{i=1}^{R}\left(1-f_{t}^{wk,i}C_{t-1}^{wk,i}\right),\\ \;U_{t}^{wk}=(U_{t-1}^{wk}+W_{t-1}^{wk}-(U_{t-1}^{wk}{}^{\circ}W_{t-1}^{wk})){}^{\circ}\psi_{t}^{wk},\\ \;\phi_{t}^{wk}=\text{SortIndiceAscending}(U_{t}^{wk}),\\ A_{t}^{wk}[\phi_{t}^{wk}[j]]=(1-U_{t}^{wk}[\phi_{t}^{wk}[j]])\prod_{i=1}^{j-1}U_{t}^{wk}[\phi_{t}^{wk}[j]]], \end{array}$$

where $\psi_{t}^{wk}\in {\mathbb{R}}^{N}$ is the result of scaling and accumulating the read weight matrix $C_{t-1}^{wk,i}$ from the previous time step using the $f_{t}^{wk,i}$ gated vector. The $\phi_{t}^{wk}\in {\mathbb{R}}^{N}$ tensor is the index tensor, sorted in ascending order by the memory region management tensor $U_{t}^{wk}\in {\mathbb{R}}^{N}$, where *N* represents the length of the memory region. Additionally, $A_{t}^{wk}\in {\mathbb{R}}^{N}$ represents the write weight of the working memory region based on dynamic addressing.

Specifically, in Equation 3, the tensor $U_{t}^{wk}$ precisely tracks the usage frequency and recency of each memory slot. A low value in $U_{t}^{wk}$ directly indicates infrequent access or prolonged non-usage. The free gate vectors ($f_{t}^{wk,i}$) from multiple read heads further modulate the retention values of memory slots, explicitly controlling the deletion of recently extracted items. Consequently, memory slots with persistently low $U_{t}^{wk}$ values, resulting from limited read/write activities over multiple consecutive time steps, are considered to have not been used for a “long time” and thus are candidates for deletion.

The method for calculating write weights based on content addressing in the working memory region is presented in Equation 4 (Graves et al., 2016; Hsin, 2016):


(4) $$\begin{array}{l} C_{t}^{wk}=\frac{exp\left(d\left(K_{t}^{wk},M_{t}^{wk}\right)\beta_{t}^{wk}\right)}{\sum exp\left(d\left(K_{t}^{wk},M_{t}^{wk}\right)\beta_{t}^{wk}\right)}, \end{array}$$

where $C_{t}^{wk}\in {\mathbb{R}}^{N}$, $\beta_{t}^{wk}\in \mathbb{R}$, $K_{t}^{wk}\in {\mathbb{R}}^{W}$, and $M_{t}^{wk}\in {\mathbb{R}}^{N\times W}$ represent the working memory region, and $d\left(u,v\right)=\frac{u\cdot v}{\left|u||v\right|}$. Here, *N* represents the length of the memory region, and *W* represents the width of the memory region.

The write algorithm for the working memory region is presented in Equation 5 (Graves et al., 2016; Hsin, 2016):


(5) $$\begin{array}{l} \begin{array}{c} W_{t}^{wk}=\gamma_{t}^{wk}\left[g_{t}^{wk}A_{t}^{wk}+\left(1-g_{t}^{wk}\right)C_{t}^{wk}\right],\\ M_{t}^{wk}=M_{t-1}^{wk}-M_{t-1}^{wk}{}^{\circ}W_{t}^{wk}{\left(E_{t}^{wk}\right)}^{T}+W_{t}^{wk}{\left(V_{t}^{wk}\right)}^{T}, \end{array} \end{array}$$

where $W_{t}^{wk}\in {\mathbb{R}}^{N}$ represents the final write weight of the working memory region, and $g_{t}^{wk}\in \left[0,1\right]$ denotes the write weight allocation gate scalar, which controls the allocation proportion of the two addressing modes in the final write. The gating scalar $\gamma_{t}^{wk}\in \left[0,1\right]$ serves to protect the data in the memory region, preserving its relative stability and preventing it from being overwhelmed by unimportant, redundant, or irrelevant information.

**Working Memory Extraction Algorithm**. In the extraction of working memory, the information most relevant to the current interactive read query signal $K_{t}^{wk,i}$ is retrieved. The extraction weighting algorithm is defined by Equation 6 as follows (Graves et al., 2016; Hsin, 2016):


(6) $$\begin{array}{l} C_{t}^{wk,i}=\frac{\exp\left(d\left(K_{t}^{wk,i},M_{t}^{wk}\right)\beta_{t}^{wk,i}\right)}{\sum \exp\left(d\left(K_{t}^{wk,i},M_{t}^{wk}\right)\beta_{t}^{wk,i}\right)}, \end{array}$$

where $C_{t}^{wk,i}\in {\mathbb{R}}^{R\times N}$, $K_{t}^{wk,i}\in {\mathbb{R}}^{R\times W}$, and $\beta_{t}^{wk,i}\in {\mathbb{R}}^{R}$, with *R* representing the total number of read operations, and *i* indicating the specific label.

The information extraction algorithm within the working memory region is defined by Equation 7 as follows:


(7) $$\begin{array}{l} R_{t}^{wk,i}={\left(M_{t}^{wk}\right)}^{T}C_{t}^{wk,i}, \end{array}$$

where $R_{t}^{wk,i}\in {\mathbb{R}}^{R\times W}$.

#### 3.2.2 Memory transformation mechanism

The DNC-based model (Graves et al., 2016; Franke et al., 2018) directly maps the output of the working memory ($R_{t}^{wk,i}$) to a linear layer. However, since the items that have been used are deleted from working memory, this leads to the loss of important information, which in turn affects both performance and robustness. We propose a memory transformation algorithm that transfers information extracted from the working memory into the long-term memory, compensating for information loss due to frequent updates and deletions in the working memory.

The algorithm for updating and extracting information in long-term memory is similar to that in working memory. The only difference is that the input in working memory originates from the controller layer, whereas the input in long-term memory originates from the working memory module. The update formula for the long-term memory region is given in Equation 8:


(8) $$\begin{array}{l} \begin{array}{c} W_{t}^{lt}=\gamma_{t}^{lt}\left[g_{t}^{lt}A_{t}^{lt}+\left(1-g_{t}^{lt}\right)C_{t}^{lt}\right],\\ B_{t}^{wk}=\overset{R}{\underset{i=1}{\prod }}R_{t}^{wk,i},\\ M_{t}^{lt}=M_{t-1}^{lt}-M_{t-1}^{lt}{}^{\circ}W_{t}^{lt}{\left(E_{t}^{lt}\right)}^{T}+W_{t}^{lt}{\left(B_{t}^{wk}\right)}^{T}, \end{array} \end{array}$$

where $M_{t}^{lt}\in {\mathbb{R}}^{N\times W}$, $B_{t}^{wt}\in {\mathbb{R}}^{W}$, and $g_{t}^{lt}\in \left[0,1\right]$ represents the long-term memory write weight allocation gate scalar, which controls the allocation proportion of the two addressing modes in the final write.

Information extraction from the memory layer integrates information from both the working memory region, $R_{t}^{wk,i}$, and the long-term memory region, $R_{t}^{lt,i}$. This calculation is given by the following equation in Equation 9:


(9) $$\begin{array}{l} \begin{array}{c} O_{t}^{m}=R\left(R_{t}^{wk,i}⊕R_{t}^{lt,i}\right), \end{array} \end{array}$$

where $O_{t}^{m}\in {\mathbb{R}}^{2RW}$, and R(·) represents the reshaping operation applied to the concatenated tensor $R_{t}^{wk,i}⊕R_{t}^{lt,i}$, transforming it into a vector of length 2*RW*.

### 3.3 Linear layer

The output of the linear layer, ŷ_*t*_, is determined by the output of the controller layer, $O_{t}^{c}$, after Dropout processing (Franke et al., 2018; Gal and Ghahramani, 2016; Srivastava et al., 2014), as well as the output of the memory layer, $O_{t}^{m}$, given by Equation 10:


(10) $$\begin{array}{l} {ŷ}_{t}=\text{Softmax}\left(\left(O_{t}^{m}⊕\text{Dropout}\left(O_{t}^{c}\right)\right)\cdot W_{t}^{o}+b_{t}^{o}\right), \end{array}$$

where ${ŷ}_{t}\in {\mathbb{R}}^{Y}$, $W_{t}^{o}\in {\mathbb{R}}^{\left(2RW+C\right)\times Y}$ is the output weight matrix, and $b_{t}^{o}\in {\mathbb{R}}^{Y}$ is the bias vector.

The detailed procedure of our MT-DNC model is shown in Algorithm 1.

**Algorithm 1:**
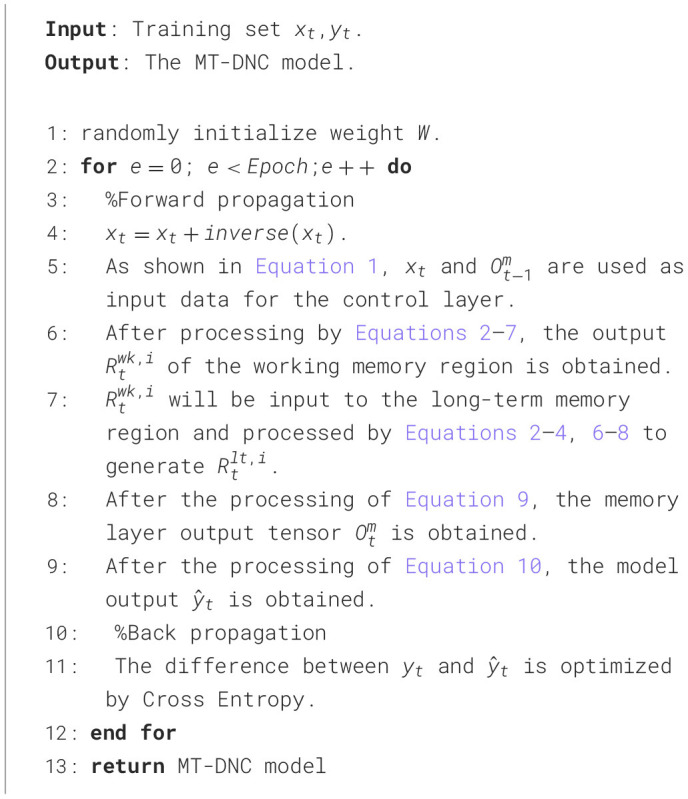
Execution algorithm for MT-DNC.

## 4 Experiments

### 4.1 The bAbI task

The bAbI^1^ is a reasoning-based text question-and-answer task (Weston et al., 2015; Kumar et al., 2016). We use the en-10k dataset for experimentation, which contains 20 sub-tasks. Each subtask contains numerous stories, with each story consisting of supporting facts, multiple questions, and their corresponding answers. The correct answers rely on one or more supporting facts. A joint training approach is employed to evaluate the text comprehension and reasoning ability of the MT-DNC model. Unlike other previous works, our method uses end-to-end training without any pre-processing of the bAbI dataset itself.

### 4.2 Training details

The bAbI question-and-answer task, comprising 20 sub-tasks, is combined into a single training session. A training sample is generated for each sub-task in the dataset, based on different stories. The detailed generation process is as follows:

1. The text sequence training samples are processed by removing digits, converting words to lowercase, removing line breaks, etc.
2. The text sequence training samples are split into lists of word sequences (including 3 punctuation marks).
3. The “answer words” in the list are replaced with “-”, and the list is then encoded into word vectors using a one-hot word vector processor. The length of the list corresponds to the length of the largest text sequence in the current batch, and shorter texts are padded with “0”. A word in the list is represented as $x_{t}\in {\mathbb{R}}^{X}$, where *X* is the length of the word vector, with a value of 159.
4. All training input samples and target samples are combined to form the training sample list.
5. 10% of the data in the training sample list is used as the validation dataset.
6. The MT-DNC model is trained for 300 epochs, with validation and testing after each epoch.

The total number of parameters in the model is 1,267,337, and the batch size is 32. The number of control layer nodes is 172, corresponding to the output dimension *C* of the control layer. Both memory regions have a length of 128 (i.e., dimension *N*) and a width of 64 (i.e., dimension *W*), with 4 read heads (i.e., *R*), 1 write head, and a dropout rate of 0.9. The learning rate is 0.0003, and the momentum value of the Rmsprop optimizer is 0.9 (Kingma and Ba, 2014). The gradient clipping value is set to 10.

### 4.3 Experimental results

To verify the effectiveness of the proposed MT-DNC model, we conducted comparison experiments with DNC, EntNet (Henaff et al., 2016), LSTM (Hochreiter and Schmidhuber, 1997), SDNC (Rae et al., 2016), BrsDNC (Franke et al., 2018), and other models on the bAbI question-and-answer task. Additionally, we evaluated the MT-DNC-DI model (a variant of our MT-DNC model without the memory transformation mechanism, where “DI” stands for Direct Independence) to assess the impact of the memory transformation algorithm on model performance. The MT-DNC-DI model employs independent memory modules, with separate regions for working memory and long-term memory, both of which receive input directly from the controller layer. Table 1 shows the average word error rate (WER) of different models under different initialized parameters.

**Table 1:** The average word error rate (WER) of different models on bAbI task (Henaff et al., 2016; Hochreiter and Schmidhuber, 1997; Rae et al., 2016; Franke et al., 2018).

**Task**	**DNC**	**EntNet**	**LSTM**	**SDNC**	**BrsDNC**	**MT-DNC-DI**	**MT-DNC**
1: 1 supporting fact	9.0 ± 12.6	0.0 ± 0.1	28.4 ± 1.5	0.0 ± 0.0	0.1 ± 0.1	0.0 ± 0.0	**0.0** **±0.0**
2: 2 supporting facts	39.2 ± 20.5	15.3 ± 15.7	56.0 ± 1.5	7.1 ± 14.6	0.8 ± 0.2	0.3 ± 0.2	**0.4** **±0.3**
3: 3 supporting facts	39.6 ± 16.4	29.3 ± 26.3	51.3 ± 1.4	9.4 ± 16.7	2.4 ± 0.6	2.8 ± 0.8	2.7 ± 0.8
4: 2 argument relations	0.4 ± 0.7	0.1 ± 0.1	0.8 ± 0.5	0.1 ± 0.1	0.0 ± 0.0	0.0 ± 0.0	**0.0** **±0.0**
5: 3 argument relations	1.5 ± 1.0	0.4 ± 0.3	3.2 ± 0.5	0.9 ± 0.3	0.7 ± 0.1	0.6 ± 0.3	**0.5** **±0.1**
6: yes/no questions	6.9 ± 7.5	0.6 ± 0.8	15.2 ± 1.5	0.1 ± 0.2	0.0 ± 0.0	0.0 ± 0.0	**0.0** **±0.0**
7: counting	9.8 ± 7.0	1.8 ± 1.1	16.4 ± 1.4	1.6 ± 0.9	1.0 ± 0.5	0.6 ± 0.3	**0.6** **±0.2**
8: lists/sets	5.5 ± 5.9	1.5 ± 1.2	17.7 ± 1.2	0.5 ± 0.4	0.5 ± 0.3	0.0 ± 0.0	**0.1** **±0.1**
9: simple negation	7.7 ± 8.3	0.0 ± 0.1	15.4 ± 1.5	0.0 ± 0.1	0.1 ± 0.2	0.0 ± 0.0	**0.0** **±0.0**
10: indefinite knowledge	9.6 ± 11.4	0.1 ± 0.2	28.7 ± 1.7	0.3 ± 0.2	0.0 ± 0.0	0.0 ± 0.0	**0.0** **±0.0**
11: basic coreference	3.3 ± 5.7	0.2 ± 0.2	12.2 ± 3.5	0.0 ± 0.0	0.0 ± 0.0	0.0 ± 0.0	**0.0** **±0.0**
12: conjunction	5 ± 6.3	0.0 ± 0.0	5.4 ± 0.6	0.2 ± 0.3	0.0 ± 0.1	0.0 ± 0.0	**0.0** **±0.0**
13: compound coreference	3.1 ± 3.6	0.0 ± 0.1	7.2 ± 2.3	0.1 ± 0.1	0.0 ± 0.0	0.0 ± 0.0	**0.0** **±0.0**
14: time reasoning	11 ± 7.5	7.3 ± 4.5	55.9 ± 1.2	5.6 ± 2.9	0.8 ± 0.7	0.0 ± 0.0	**0.0** **±0.0**
15: basic deduction	27.2 ± 20.1	3.6 ± 8.1	47.0 ± 1.7	3.6 ± 10.3	0.1 ± 0.1	0.0 ± 0.0	**0.0** **±0.0**
16: basic induction	53.6 ± 1.9	53.3 ± 1.2	53.3 ± 1.3	53.0 ± 1.3	52.6 ± 1.6	49.1 ± 0.9	**38.8** **±11.1**
17: positional reasoning	32.4 ± 8	8.8 ± 3.8	34.8 ± 4.1	12.4 ± 5.9	4.8 ± 4.8	4.2 ± 0.9	**0.6** **±1.1**
18: size reasoning	4.2 ± 1.8	1.3 ± 0.9	5.0 ± 1.4	1.6 ± 1.1	0.4 ± 0.4	0.4 ± 0.2	**0.0** **±0.0**
19: path finding	64.6 ± 37.4	70.4 ± 6.1	90.9 ± 1.1	30.8 ± 24.2	0.0 ± 0.0	0.0 ± 0.0	0.4 ± 0.8
20: agents motivation	0.0 ± 0.1	0.0 ± 0.0	1.3 ± 0.4	0.0 ± 0.0	0.1 ± 0.1	0.0 ± 0.0	**0.0** **±0.0**
Mean WER:	16.7 ± 7.6	9.7 ± 2.6	27.3 ± 0.8	6.4 ± 2.5	3.2 ± 0.5	2.9 ± 0.0	**2.2** **±0.5**
Failed Tasks (>5%):	11.2 ± 5.4	5.0 ± 1.2	17.1 ± 1.0	4.1 ± 1.6	1.4 ± 0.5	1.4 ± 0.4	**1.0** **±0.0**

The bold values indicate the best performance in the comparison experiments.

According to the experimental results, the MT-DNC model achieves a lower average error rate (2.2% mean WER) compared to other models, particularly the representative BrsDNC model, which demonstrates superior performance with a mean WER of 3.2% on the 20 bAbI sub-tasks under joint training. Specifically, for the 14*th*, 15*th*, and 18*th* sub-tasks, all other methods produce errors, while our method achieves an error rate of 0%. For the 16*th* and 17*th* sub-tasks, our method significantly reduces the error rate by 13.8% and 4.2%, respectively, compared to the BrsDNC model. Additionally, we counted the number of failed tasks (those with more than 5% errors) across the 20 sub-tasks, as shown in the last row of Table 1. Our method has only one failed task and outperforms other methods, significantly surpassing the DNC (with 11 failed tasks) and LSTM (with 17 failed tasks) models.

**Figure 2:**
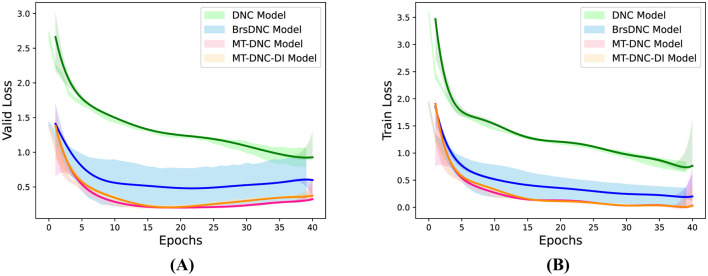
Validation loss **(A)** and training loss **(B)** of DNC, BrsDNC, MT-DNC-DI and MT-DNC. The horizontal axis represents the number of Epochs and the vertical axis represents the change of loss.

Figure 2 illustrates the loss trends of different models during validation (Figure 2A) and training (Figure 2B) processes. As shown, the MT-DNC model demonstrates lower loss, higher performance, and faster convergence compared to the DNC and BrsDNC models. Furthermore, the variance of the learning curves in Figures 2A, B indicates that our method is more stable, with minimal fluctuations, while the BrsDNC model exhibits significant instability and fluctuating learning processes. Overall, our MT-DNC model improves convergence speed and performance while maintaining superior stability.

### 4.4 Ablation study

To further analyze the validity of our proposed model, we conducted a series of ablation experiments. The main innovation of our model lies in the introduction of long-term memory and the memory transformation algorithm. In the MT-DNC model, the long-term memory module receives input from the working memory module through the memory transformation algorithm. To verify the effectiveness of the memory transformation mechanism, we compared the performance of MT-DNC and MT-DNC-DI. In the MT-DNC-DI model, the long-term memory module receives input directly from the controller layer (with different parameters from the working memory module). From Table 1 and Figure 2, we observe that MT-DNC achieves superior performance compared to MT-DNC-DI, both in terms of WER on each sub-task and in terms of average WER. Additionally, the MT-DNC-DI model performs better and exhibits lower loss compared to DNC, BrsDNC, and other models, indicating that the long-term memory itself contributes positively to model performance, while the memory transformation mechanism further enhances it.

**Figure 3:**
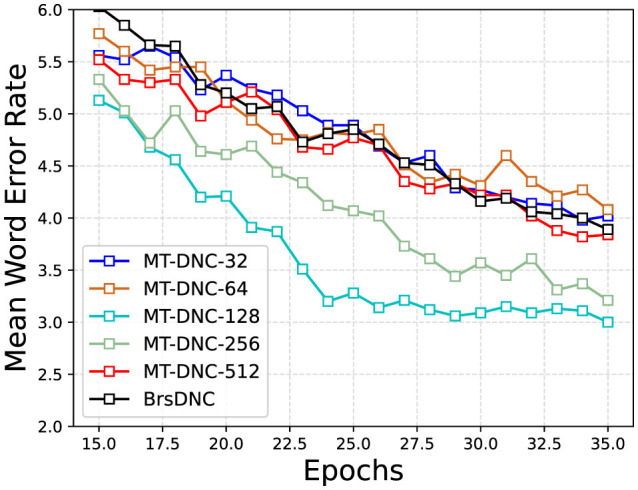
Mean Word Error Rate of MT-DNC-32, MT-DNC-64, MT-DNC-128, MT-DNC-256, MT-DNC-512, BrsDNC. The horizontal coordinate represents the number of Epochs and the vertical coordinate represents the changing of Mean Word Error Rate.

We also analyzed the effect of storage space in long-term memory and working memory on the experimental results. Figure 3 illustrates the changes in mean WER during the learning process at different memory space sizes. We compared these results with the changes in mean WER of the BrsDNC model (black line in Figure 3). The experimental results reveal that when the memory space is too small (e.g., 32 or 64), the performance of the model is negatively affected. Our model achieves comparable performance to the BrsDNC model under very small memory spaces (32 and 64), despite the BrsDNC model using a larger memory space of 128. However, our MT-DNC model significantly outperforms the BrsDNC model at memory space lengths of 128 and 256. Furthermore, we found that excessive memory space (e.g., 512) does not improve performance and instead leads to performance degradation. Overall, our model is robust and adaptable to different memory space lengths, but overly small or overly large memory spaces negatively impact performance compared to the most appropriate length.

## 5 Conclusion

In this paper, inspired by the memory transformation mechanism of the human brain, we propose the MT-DNC model, a coordinated framework with two memory modules: working memory and long-term memory. By establishing a connection between the working memory and the long-term memory, this model alleviates some of the challenges faced by DNCs. Specifically, as the amount of information in the memory region increases, the effectiveness of information retrieval and training efficiency improve, significantly impacting the model's convergence rate and final performance.

Nonetheless, several promising directions remain for future research. In particular, integrating the MT-DNC architecture with Transformer-based models is a key area of ongoing exploration. This hybrid approach aims to combine the structured, interpretable memory dynamics of MT-DNC with the powerful parallel processing capabilities of Transformers. By leveraging Transformer's inherent parallelism, the integrated model is expected to overcome the current limitations of sequential memory operations in DNC-based architectures, thereby improving computational efficiency and scalability.

## Data Availability

Publicly available datasets were analyzed in this study. This data can be found at: https://github.com/Brain-Cog-Lab/MTDNC.

## Footnotes

^1^ https://research.facebook.com/downloads/babi/

## Appendix A

### Detailed Derivation of the Memory State Dimension ( S_*t*_)

The dimension *S*_*t*_ = (2*R*+6)*W*+6+4*R* is obtained by explicitly partitioning the output of the controller into signals required by the MT-DNC memory module operations. Below is the intuitive and step-by-step derivation aligned with the source code:

1. **Working and Long-term Memory Writing Signals**

- Write keys for working and long-term memory: Each with dimension *W*, totaling 2*W*.
- Write strengths (scalars) for both memories: 2 signals, each dimension 1, totaling 2.
- Erase vectors for both memories: Each with dimension *W*, totaling 2*W*.
- Write vectors for both memories: Each with dimension *W*, totaling 2*W*.
- Allocation gates (scalars) for both memories: 2 signals, dimension 1 each, totaling 2.
- Write gates (scalars) for both memories: 2 signals, dimension 1 each, totaling 2.

**Subtotal:** 6*W*+6

2. **Reading Signals (with multiple read heads** *R***)**

- Read keys for working and long-term memories: Each memory has *R* heads, each head dimension *W*, totaling 2*RW*.
- Read strengths for working and long-term memories: Each memory has *R* read heads, each head a scalar, totaling 2*R*.
- Free gates for working and long-term memories: Each memory has *R* read heads, each a scalar, totaling 2*R*.

**Subtotal:** 2*RW*+4*R*

3. **Combine All Components**

$$S_{t}=\left(6W+6\right)+\left(2RW+4R\right)=\left(2R+6\right)W+6+4R$$

This derivation matches precisely the dimensional partitioning provided in the implementation code as follows:

 
  write_keys: [W]
  write_keys_sec: [W]                     # 2W
  write_strengths: [1]
  write_strengths_sec: [1]                # 2
  erase_vector: [W]
  erase_vector_sec: [W]                   # 2W
  write_vector: [W]
  write_vector_sec: [W]                   # 2W
  alloc_gates: [1]
  alloc_gates_sec: [1]                    # 2
  write_gates: [1]
  write_gates_sec: [1]                    # 2
  
  # Total so far: 6W + 6
  
  read_keys: [R x W]
  read_keys_sec: [R x W]                  # 2RW
  read_strengths: [R]
  read_strengths_sec: [R]                 # 2R
  free_gates: [R]
  free_gates_sec: [R]                     # 2R
  
  # Total addition: 2RW + 4R
  
  # Final total: (2R + 6)W + 6 + 4R
 

Each component is explicitly represented and corresponds exactly to the signals used by the memory operation algorithms (writing, erasing, reading, gating), facilitating clear understanding and precise reproducibility of the MT-DNC architecture.
